# Dosimetric Comparison between Three-Dimensional Magnetic Resonance Imaging-Guided and Conventional Two-Dimensional Point A-Based Intracavitary Brachytherapy Planning for Cervical Cancer

**DOI:** 10.1371/journal.pone.0161932

**Published:** 2016-09-09

**Authors:** Juan Ren, Wei Yuan, Ruihua Wang, Qiuping Wang, Yi Li, Chaofan Xue, Yanli Yan, Xiaowei Ma, Li Tan, Zi Liu

**Affiliations:** 1 Department of Radiotherapy, Oncology Center, First Affiliated Hospital of Xi’an Jiaotong University, Xi’an, Shaanxi, P.R. China; 2 Department of Imaging, First Affiliated Hospital of Xi’ an Jiaotong University, Xi’an, Shaanxi, P.R. China; 3 Medical School, Xi’ an Jiaotong University, Xi’an, Shaanxi, P.R. China; North Shore Long Island Jewish Health System, UNITED STATES

## Abstract

**Objective:**

The purpose of this study was to comprehensively compare the 3-dimensional (3D) magnetic resonance imaging (MRI)-guided and conventional 2-dimensional (2D) point A-based intracavitary brachytherapy (BT) planning for cervical cancer with regard to target dose coverage and dosages to adjacent organs-at risk (OARs).

**Methods:**

A total of 79 patients with cervical cancer were enrolled to receive 2D point A-based BT planning and then immediately to receive 3D planning between October 2011 and April 2013 at the First Hospital Affiliated to Xi’an Jiao Tong University (Xi’an, China). The dose-volume histogram (DVH) parameters for gross tumor volume (GTV), high-risk clinical target volume (HR-CTV), intermediate-risk clinical target volume (IR-CTV) and OARs were compared between the 2D and 3D planning.

**Results:**

In small tumors, there was no significant difference in most of the DVHs between 2D and 3D planning (all p>0.05). While in big tumors, 3D BT planning significantly increased the DVHs for most of the GTV, HR-CTV and IR-CTV, and some OARs compared with 2D planning (all P<0.05). In 3D planning, DVHs for GTV, HR-CTV, IR-CTV and some OARs were significantly higher in big tumors than in small tumors (all p<0.05). In contrast, in 2D planning, DVHs for almost all of the HR-CTV and IR-CTV were significantly lower in big tumors (all p<0.05). In eccentric tumors, 3D planning significantly increased dose coverage but decreased dosages to OARs compared with 2D planning (p<0.05). In tumors invading adjacent tissues, the target dose coverage in 3D planning was generally significantly higher than in 2D planning (P<0.05); the dosages to the adjacent rectum and bladder were significantly higher but those to sigmoid colon were lower in 3D planning (all P<0.05).

**Conclusions:**

3D MRI image-guided BT planning exhibits advantages over 2D planning in a complex way, generally showing advantages for the treatment of cervical cancer except small tumors.

## Introduction

Cervical cancer ranks the fourth most common cancer in women. Radiotherapy, including external beam radiotherapy (EBRT) and intracavitary brachytherapy (BT), is a major and important treatment measure for patients with bulky cancer of the cervix; among which, intracavitary BT plays key roles in the effective treatment. Until now, BT for cervical cancer is still widely relied on the conventional two-dimensional (2D) X-ray radiographs, especially in developing countries such as China, which has remained relatively unchanged for many years. The prescription of dose for 2D X-ray radiograph-based BT is relied on point A with a fixed reference point 2 cm lateral to the applicator [1], regardless of the individual patient’s anatomy or tumor characteristics (e.g. size, type), which thus might yield in inadequate dosage calculation and target coverage [2]. In the meanwhile, the adjacent organs-at risk (OARs) such as rectum and bladder can receive higher than necessary dosages when the dose is prescribed to a fixed point, which contributes to the higher risk of serious radiotherapy complications. This is crucial because of the close proximity of these organs to cervix and associated toxicities [3, 4].

Adequate coverage of tumor target results in well local control and outcomes, which is vital to the BT for cervical cancer,. Given the defaults of 2D X-ray BT planning, three-dimensional (3D) imaging (e.g. computed tomography [CT] and magnetic resonance imaging [MRI])-guided intracavitary BT planning for cervical cancer has been developed [2, 5–7]. This involves the dose prescription according to a 3D target tumor volume instead of a fixed reference point, thereby providing better dosage optimization. On the basis of the determination of actual disease extent, 3D imaging—based BT adequately treats tumors with minimal complications in normal tissues as well as predicts the treatment outcomes [7–9].

Reportedly, 3D CT planning is superior to the conventional 2D planning as the latter overestimates the tumor dosages but underestimates the OAR dosages [10, 11]. Recently, 3D MRI-guided BT planning for cancer of the cervix has been developed, which has the potential to optimize the primary tumor dosimetry and reduce the dosages to critical normal tissues [2, 7, 12]. For primary tumors and adjacent soft tissues in the pelvis, MRI appears to be better than CT for more accurate estimation of tumor coverage [13, 14]. Therefore, 3D MRI-guided BT is recently more recommended in the cervical cancer treatment.

Until now, although the 2D and 3D BT planning for cervical cancer has been compared, such analysis still needs to be further verified in detail (e.g. based on the tumor size, morphology characters, invasion into the adjacent organs). The purpose of this study was to comprehensively compare the tumor dose coverage and dosages to adjacent OARs between the 3D MRI- and 2D point A-based BT planning for patients with cervical cancer as well as determine the potential benefits of 3D image-guided plan optimization. This study showed that compared with 2D point A-based BT planning, 3D MRI-based planning generally significantly increased dose coverage in big tumors, eccentric tumors and tumors invading adjacent tissues, while diversely affected the dosages to OARs. The difference in the target dose coverage and dosages to OARs between small and big cervical tumors was different in 2D and 3D BT planning. This study indicates that 3D MRI image-guided BT planning exhibits advantage over 2D point A-based planning in a complex way, generally showing advantages in the treatment of cervical cancer except small tumors.

## Materials and Methods

### Patients

This was a prospective study. A total of 79 patients with cervical cancer were enrolled to be first treated with CT-based external beam radiation therapy (EBRT) using either 3D conformal radiotherapy or intensity- modulated radiation therapy, and then with high-dose rate (HDR)-BT between October 2011 and April 2013 at the First Hospital Affiliated to Xi’an Jiao Tong University (Xi’an, China). For HDR-BT, all patients underwent both 2D and 3D planning. Since the radiotherapy for the patients in this study was still based on the conventional 2D BT planning data and that 2D BT planning takes much shorter time than 3D planning, 2D planning was carried out prior to 3D BT planning. Patients were treated with point A-based 2D BT planning (24 Gy in 4 fractions), immediately followed by 3D BT planning with the dosage of 24 Gy in 4 fractions and the dosage rate of more than 12 Gy/h. Cervical cancer was confirmed by histological examination on biopsy. All patients were treated for curative intent and disease was staged according to the International Federation of Gynecology and Obstetrics (FIGO) system. This study was formally approved by the Ethic Committees of the First Hospital Affiliated to Xi’an Jiao Tong University. Written informed consent was obtained from the patients.

### MRI examination

MRI examination was performed initially before the start of any treatment to determine the loco-regional disease size and extent using a MRI machine (MAETE, 1.5T, Philips Health Care, Andover, MA, USA), with the MRI-compatible intrauterine applicator in place and inserted into the catheters to locate the intrauterine and vaginal positions. T1- and T2-weighted sequence in the para-axial, para-coronal and para-sagittal planes (parallel to applicators) were acquired. A second MRI examination was then performed after the completion of EBRT and immediately before BT. Axial (perpendicular to body) and sagittal T2- weighted, turbo spin echo MRIs were conducted. Parameters for axial images were: field of view 35 cm, repetition time 4600 ms, excitation time 90 ms, slice thickness 3 mm, gap thickness 0 mm, and matrix 512×512. Parameters for sagittal images were: field of view 38 cm, repetition time 3600 ms, excitation time 90 ms, slice thickness 3 mm, gap thickness 0 mm, and matrix 512×512.

Immediately after MRI scanning, orthogonal radiographs were taken to check the image reconstruction of the applicator. Based on the International Commission on Radiation Units (IRCU), reference points were registered on the MRIs, including right and left points A, right and left pelvic wall points, ICRU bladder and rectal points, and one additional point (1.5 cm cranial to the ICRU bladder point).

### Delineation of gross tumor volume (GTV), high-risk clinical target volume (HR-CTV), intermediate-risk clinical target volume (IR-CTV) and walls of OARs

GTV, HR-CTV and IR-CTV, as well as rectal, sigmoid and bladder walls were defined and delineated on each MRI image following Gynaecological GEC-ESTRO guidelines [15, 16] by an experienced radiation oncologist who was blind to this study. GTV included macroscopic tumor extension area prior to the first afterloading and each subsequent BT as delineated based on the clinical gynecological and MRI examinations. HR-CTV, with a major risk of local recurrence for residual macroscopic tumor, included the whole cervix and the presumed extracervical tumor expansion area upon the BT. Generally, HR-CTV comprised GTV and an upper safety margin of <15 mm and left and right safety margin of 5 mm, respectively. IR-CTV has a major risk of local recurrence in areas corresponding to initial macroscopic extent of disease with at most residual microscopic disease at time of BT. IR-CTV was contoured as a volume with the HR-CTV plus a safe margin of 5–15 mm. Amount of safety margin depended on the tumor size and location, potential tumor spread directions, tumor regression and treatment strategy. According to the HR-CTV of tumors, the patients were divided into small tumor (HR-CTV< 30 cm^3^) group and big tumor (HR-CTV≥ 30 cm^3^) group.

### EBRT and intracavitary BT

For EBRT, the prescription dosage (50.4 Gy in 28 fractions) was delivered to the pelvis or pelvis para-aortic region, depending on the clinical stages of diseases. Additional boost dosage of 5.4 Gy in 3 fractions was delivered to the parametrium.

BT was initiated after the completion of EBRT using Fletcher CT-MRI-compatible applicator (Part number 189.730, Nucletron Systems, Veenendaal, Netherlands). Ovoid tube and intrauterine tube were assembled and placed according to the patient vaginal anatomy, and adapted to the tumor shape, size, etc. MRI examination was performed following the routine protocols.

### 2D point A-based intracavitary BT planning

The cervical os position (at the Smit sleeve distal end), tandem and ring applicator were determined on MRI images, and were used as an origin of 2D coordinates. Point A (2 cm above and 2 cm lateral to the cervical os) and Point T (1 cm above and 1 cm lateral to the cervical os) were marked on the corresponding MRI images. During the afterloading, the length of radiation source through the intrauterine tube was determined according to the lengths of the uterine cavity, cervical os and lesion. The intrauterine tube in the applicator had a segment (3 cm in length) which could be used to pass through the radiation source; and a section of 1 cm long from the middle of this segment was selected to pass through the radiation source. Measurement was performed from cervical os position to the ring applicator plane as well as from the tip of the tandem and ring to the first dwell position of the dummy marker. The standard loading pattern of source dwell positions was put into the planning system. Radiation prescription (6 Gy each time) was given to point A. Applicator reconstruction for 2D planning was performed by digitizing source tracks, and was then input into the treatment planning system.

### 3D MRI image-guided intracavitary BT planning

The cervical os position was determined on MRI images and was also used as an origin of 3D coordinates. The tandem and ring applicator were identified on MRI images. The treatment planning system provided additional 3D coordinates of applicators and source path positions as well as graphic images of the applicators. The target was determined from the pretreatment MRI scans in combination with clinical examination. Radiation prescription (6 Gy each time) was given to HR-CTV. 3D treatment planning was generated using the Nucletron PLATO Brachytherapy Planning System (Nucletron Systems, Veenendaal). After isodose lines were generated from the standard loading pattern, optimization was performed by changing dwell weightings or isodose lines in order to adjust dosage to the target and monitor the OAR dosages. Dose-volume histograms (DVHs) for OARs (sigmoid colon, rectum, and bladder) were calculated to evaluate the dosages to OARs. The minimum dosages delivered to 50, 90, 95 and 100% of the target volume were calculated as D50, D90, D95, and D100, respectively for all individual fractions. The minimum dosages to the highest irradiated areas of 0.1, 1, 2 and 5 cm^3^ in OARs were recorded as D0.1cm^3^, D1cm^3^, D2cm^3^ and D5cm^3^, respectively. Tumor volume receiving 100% of the prescription dosages was recorded as V100. Intensity of modulated radiation therapy planning was optimized to ensure 95% isodose curve should cover 95% volume of HR-CTV.

The total dosages from EBRT and BT were summated and normalized to a biologically equivalent dosage of 2 Gy per fraction (EQD2) using the linear quadratic model with α/β of 10 Gy for tumors and α/β of 3 Gy for OARs. The planned EQD2 for HR-CTV was between 88–90 Gy, whereas the dose for 2 mL of rectum, bladder, and sigmoid was≤75Gy, ≤85 Gy, and ≤75 Gy, respectively [7, 16, 17].

### Statistical analysis

All tumor and OAR DVH parameters were examined to ensure approximately normal distributions. The results were calculated as mean± standard deviation. The DVHs for the tumors and OARs were compared between 3D and 2D BT planning using the pair t-test; and for the comparison between the small and big tumors in 2D and 3D BT planning, respectively, unpaired t-test was used. SPSS 16.0 version software (SPSS Inc., Chicago, IL, USA) was used for statistical analysis, and a p-value of <0.05 was considered statistical significant.

## Results

### Patients’ characteristics

Seventy-nine patients were treated in this study. Patients’ characteristics were summarized in Table 1. The mean age of the patients was 51.9 (range 32–72) years, the main histological type was squamous cell carcinoma (77/79, 97.5%), and the main stages were FIGO stage IIb (42/79, 51.9%) and IIIb (29/79, 36.7%). Twenty patients had big tumors, and 59 had small tumors. Among the tumors, 15 were eccentric, and 7 invaded the adjacent tissues. In all patients, the mean tumor volume at diagnosis was 32.33 (range 10–136) cm^3^, mean GTV was 1.99 (range 0–13.6) cm^3^, mean HR-CTV was 37.54 (range 16.51–91.52) cm^3^, and mean IR-CTV was 115.57 (range 58.95–209.20) cm^3^ (Table 1).

**Table 1:** Patient basic characteristics.

Characteristics	Value
**Eligible patients (n)**	79
**Age (year)**	51.9 (range 32–72)
**Histological type (n/%)**	79
** Squamous cell carcinoma**	77 (97.5%)
** Adeno-squamous**	2 (2.5%)
**FIGO stage (n/%)**	
** II a**	2 (2.5%)
** II b (Endogenous grown)**	8 (10.1%)
** II b (Exogenous grown)**	33 (41.8%)
** III a**	1 (1.3%)
** III b (Endogenous grown)**	8 (10.1%)
** III b (Exogenous grown)**	21 (26.6%)
** IV a**	1 (1.3%)
** IV b**	5 (6.3%)
**Tumor volume at diagnosis (cm**^**3**^**)**	32.33 (range 10–136)
**GTV (cm**^**3**^**)**	1.99 (range 0–13.6)
**HR-CTV (cm**^**3**^**)**	37.54 (range 16.51–91.52)
**IR-CTV (cm**^**3**^**)**	115.57 (range 58.95–209.20)
**EBRT dosage (Gy)**	50.4 Gy in 28 fractions
**Hyperthermia treatment**	14 cases
**BT dosage (Gy)**	24 Gy in 4 fractions
**BT dosage rate (Gy/h)**	> 12

GTV: gross tumor volume, HR-CTV: high-risk clinical target volume, IR-CTV: intermediate-risk clinical target volume, EBRT: external beam radiotherapy, BT: brachytherapy.

### The dosimetric comparison between 2D and 3D BT planning for small and big tumors, respectively

In small tumors, D100 for GTV and D50 for HR-CTV were significantly higher in 3D planning than those in 2D BT planning, respectively (both p<0.05). However, there was no significant difference in other DVH parameters for GTV, HR-CTV and IR-CTV between 2D and 3D BT planning (all p>0.05). In addition, there was no significant difference in the volume dosages (D0.1cm^3^, D1cm^3^, D2cm^3^, D5cm^3^) and maximum dose (Dmax) to OARs including rectum, bladder and sigmoid colon between 3D and 2D BT planning (all p>0.05) (Table 2).

**Table 2:** Dosimetric comparison between 2D and 3D BT planning for small and big tumors, respectively.

	**Plan**	**D50 (%)**	**D90 (%)**	**D95 (%)**	**D100 (%)**	**V100 (cm**^**3**^**)**
**Small tumors (n = 59)**						
**GTV**	**2D planning**	262.51 ±2.31	182.59 ±1.78	170.31 ±1.37	134.38 ±1.21	1.74 ±0.00
	**3D planning**	242.85±2.31	190.20±1.82	176.25±1.63	156.34±1.49*	1.74±0.00
**HR-CTV**	**2D planning**	183.39 ±1.78	111.04 ±1.02	98.58±0.89	68.96 ±0.49	21.56 ±0.00
	**3D planning**	213.54±2.02*	112.93±1.02	98.87±0.94	66.34±0.48	22.20±0.00
**IR-CTV**	**2D planning**	118.78 ±1.01	64.31 ±0.43	56.63 ±0.46	39.43±0.28	49.91±0.01
	**3D planning**	119.29±1.01	60.66 ±0.53	54.14±0.41	38.34±0.27	51.90±0.03
**Big tumors (n = 20)**						
**GTV**	**2D planning**	262.96 ±2.45	183.80±1.70	170.11±1.61	139.70±1.20	2.37±0.00
	**3D planning**	263.31±2.51	218.49±2.07*	209.19±2.01*	185.21±1.76*	2.56±0.00*
**HR-CTV**	**2D planning**	165.83±1.52	91.47±0.89	80.49±0.71	56.68±0.41	36.98±0.02
	**3D planning**	227.03±2.15*	121.27±1.02*	104.61±1.01*	72.76±0.50*	44.29±0.02*
**IR-CTV**	**2D planning**	99.92 ± 0.91	56.14±0.49	49.71±0.36	35.51±0.32	65.47±0.03
	**3D planning**	129.08±1.15*	69.16±0.51*	61.44±0.41*	44.15±0.31	89.93±0.73*
		**D0.1cm**^**3**^ **(Gy)**	**D1cm**^**3**^ **(Gy)**	**D2cm**^**3**^ **(Gy)**	**D5cm**^**3**^ **(Gy)**	**Dmax (Gy)**
**Small tumors (n = 59)**						
**Rectum**	**2D planning**	4.12 ± 0.00	3.65 ± 0.00	3.45 ± 0.00	3.12 ± 0.00	4.71 ± 0.01
	**3D planning**	4.29±0.00	3.71±0.00	3.47±0.00	3.10±0.00	4.88±0.01
**Bladder**	**2D planning**	5.34 ± 0.00	4.78 ± 0.01	4.35 ± 0.00	4.03 ± 0.00	5.90 ± 0.01
	**3D planning**	5.52±0.00	4.78±0.01	4.43±0.00	3.84±0.00	6.13±0.01
**Sigmoid colon**	**2D planning**	4.56 ± 0.00	4.23 ± 0.00	3.99 ± 0.00	3.59 ± 0.00	5.09 ± 0.00
	**3D planning**	4.46±0.00	3.91±0.00	3.65±0.00	3.24±0.00	5.02±0.00
**Big tumors (n = 20)**						
**Rectum**	**2D planning**	4.12 ± 0.01	3.62 ± 0.01	3.48 ± 0.00	3.13 ± 0.00	4.69 ± 0.01
	**3D planning**	5.52±0.01*	4.71±0.01	4.37±0.00	3.85±0.00	6.33±0.01*
**Bladder**	**2D planning**	5.39 ± 0.01	4.75 ± 0.00	4.32 ± 0.00	4.02 ± 0.00	5.87 ± 0.01
	**3D planning**	6.08±0.01	5.17±0.00	4.79±0.00	4.19±0.00	6.81±0.01*
**Sigmoid colon**	**2D planning**	4.95 ± 0.00	4.51 ± 0.00	4.21 ± 0.00	3.83 ± 0.00	5.55 ± 0.01
	**3D planning**	6.42±0.00*	5.40±0.00	5.05±0.00	4.42±0.00	7.31±0.01*

2D: 2-dimensional, 3D: 3-dimensional, BT: brachytherapy, GTV: gross tumor volume, HR-CTV: high-risk clinical target volume, IR-CTV: intermediate-risk clinical target volume, 2D: 2-dimensional; 3D: 3-dimensional.

*P<0.05 compared with 2D planning.

In big tumors, except D50 for GTV and D100 for IR-CTV, all other DVH parameters for GTV, HR-CTV and IR-CTV were significantly higher in 3D planning than in 2D planning (all p<0.05). D0.1 cm^3^, D1cm^3^, D2cm^3^, D5cm^3^, and Dmax for OARs (including rectum, bladder, sigmoid colon) were all higher in 3D BT planning than in 2D BT planning. Particularly, D0.1cm^3^ and Dmax for rectum and sigmoid colon respectively and Dmax for bladder were significantly higher in 3D planning (all p <0.05, Table 2).

### Dosimetric difference between small and big tumors in 2D and 3D BT planning, respectively

We then further observed the dosimetric difference between small and big tumors in 2D and 3D planning, respectively. S1 Table showed that in 3D planning, D50, D90, D95, and D100 for GTV, HR-CTV and IR-CTV, respectively in big tumors were all significantly higher than those in small tumors (all p<0.05). In addition, D0.1cm^3^, D2cm^3^ and Dmax for sigmoid colon, and D0.1cm^3^ and Dmax for rectum and bladder, respectively were significantly higher in big tumors than in small tumors (all p<0.05).

In 2D planning, D50, D90, D95, and D100 for HR-CTV as well as D50, D90, and D95 V100 for IR-CTV in big tumors were all significantly lower than in small tumors (all p<0.05). In addition, Dmax for sigmoid colon was significantly higher in big tumors than in small tumors (p<0.05, S1 Table).

### Dosimetric difference between 2D and 3D BT planning for eccentric tumors

**Fig 1:**
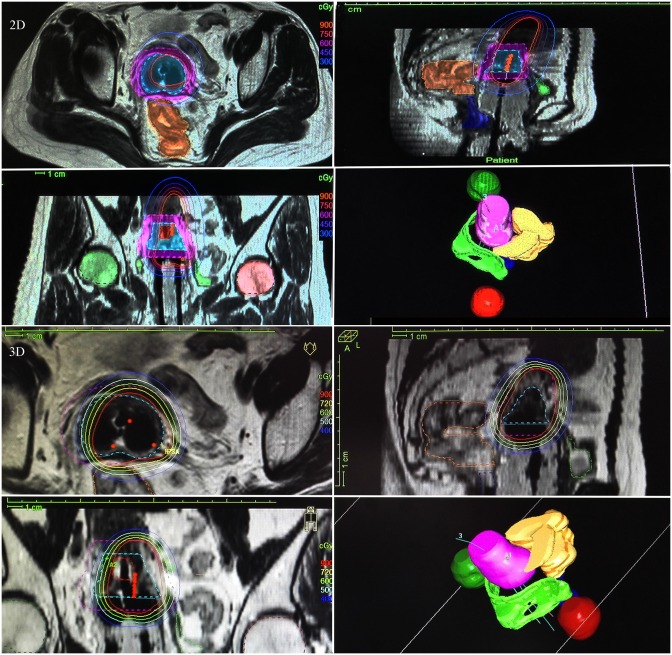
Isodose curves of 2D and 3D planning in a case with eccentric tumor. The upper 4 images were made by a 2D point A-based brachytherapy (BT) planning and the lower 4 by a 3D MRI-guided BT planning. 2D, 3D upper left: crosscut view; 2D, 3D upper right: mid-sagittal view; 2D, 3D lower left: coronal view; 2D, 3D lower right: 3D stereo diagram. The red dashed line showed gross tumor volume (GTV), cyan-blue dashed line showed high-risk clinical target volume (HR-CTV), and purple dashed line showed intermediate-risk clinical target volume (IR-CTV). Green, dark green, dark red, orange brown and dark blue area showed bladder, left femur, right femur, intestine, and rectum, respectively.

Fig 1 showed a typical case with eccentric tumor deviating to the right side. In 2D planning, the shape and target coverage of the isodose curve completely depended on the location of the BT applicator instead of the actual location, shape and size of the tumor. In contrast, with the BT dosage prescribed to the tumor volume, the isodose curve of 3D planning was optimized through intensity modulation according to the actual shape of delineated HR-CTV, thus making the isodose curve maximally cover the whole tumor (Fig 1).

Table 3 showed that except D100 and V100 for GTV and D95 for IR-CTV, all other DVHs for GTV, HR-CTV and IR-CTV were significantly higher in 3D planning than in 2D planning (all p<0.05). D0.1cm^3^, D1cm^3^, D2cm^3^, D5cm^3^ and Dmax for bladder and sigmoid colon, respectively were all significantly lower in 3D planning (all P<0.05, Table 3).

**Table 3:** Dosimetric comparison between 2D vs 3D BT planning for eccentric tumors (n = 15).

	**BT planning**	**D50 (%)**	**D90 (%)**	**D95 (%)**	**D100 (%)**	**V100 (cm^3^)**
**GTV**	**2D planning**	256.11±2.78	166.58±1.73	156.74±1.67	138.00±1.44	1.66±0.01
	**3D planning**	316.41±3.23*	199.84±2.12*	185.84±1.98*	144.11±1.67	1.66±0.01
**HR-CTV**	**2D planning**	177.13±1.81	95.69±1.12	86.17±0.92	60.14±0.78	22.94±0.02
	**3D planning**	255.48±2.48*	113.16±1.34*	99.49±1.02*	74.75±0.89*	24.86±0.02*
**IR-CTV**	**2D planning**	119.93±1.23	61.22±0.78	53.47±0.61	37.00±0.44	57.74±0.03
	**3D planning**	142.10±1.45*	64.77±0.83*	55.77±0.59	42.26±0.52*	66.39±0.04*
		**D0.1cm**^**3**^ **(Gy)**	**D1cm**^**3**^ **(Gy)**	**D2cm**^**3**^ **(Gy)**	**D5cm**^**3**^ **(Gy)**	**Dmax(Gy)**
**Rectum**	**2D planning**	1.59±0.00	1.31±0.00	1.18±0.00	0.90±0.00	2.28±0.00
	**3D planning**	1.59±0.00	1.31±0.00	1.18±0.00	0.87±0.00	2.28±0.00
**Bladder**	**2D planning**	4.16±0.00	3.04±0.00	2.71±0.00	2.12±0.00	5.16±0.01
	**3D planning**	2.98±0.00*	2.52±0.00*	2.27±0.00*	1.79±0.00*	3.60±0.00*
**Sigmoid colon**	**2D planning**	6.47±0.0167	5.60±0.01	5.10±0.01	4.22±0.00	7.32±0.01
	**3D planning**	4.55±0.01*	3.74±0.00*	3.37±0.00*	2.72±0.00*	5.52±0.01*

BT: brachytherapy, 2D: 2-dimensional, 3D: 3-dimensional, GTV: gross tumor volume, HR-CTV: high-risk clinical target volume, IR-CTV: intermediate-risk clinical target volume.

*P<0.05 compared with 2D BT planning.

### Dosimetric difference between 2D and 3D BT planning for tumors invading adjacent tissues

**Fig 2:**
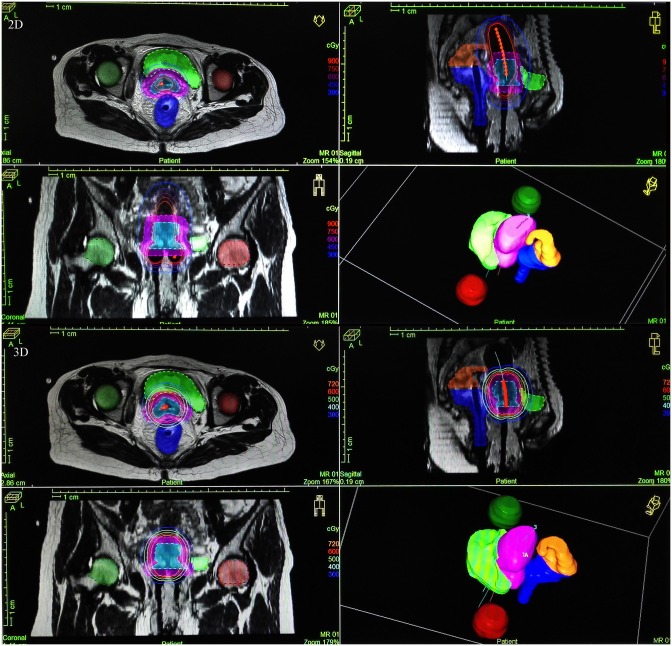
Isodose curves of 2D and 3D planning in a case with tumor invading adjacent tissues. The upper 4 images were made by a 2D point A-based brachytherapy (BT) planning and the lower 4 by a 3D MRI-guided BT planning. 2D, 3D upper left: crosscut view; 2D, 3D upper right: mid-sagittal view; 2D, 3D lower left: coronal view; 2D, 3D lower right: 3D stereo diagram. The red dashed line showed gross tumor volume (GTV), cyan-blue dashed line showed high-risk clinical target volume (HR-CTV), and purple dashed line showed intermediate-risk clinical target volume (IR-CTV). Green, dark green, dark red, orange brown and dark blue area showed bladder, left femur, right femur, intestine, and rectum respectively.

Fig 2 showed for a typical case with cervical tumor invading the bladder and ureter tissues, the volume coverage of the isodose curve was significantly more in 3D planning than in 2D planning. For patients with tumors invading adjacent tissues (e.g. bladder and rectum), all the invaded tissue volumes were included in the target volume determination (GTV, HR-CTV and IR-CTV). Table 4 showed that 3D BT planning generally provided more target dose coverage. For GTV, the D50, D90, D95 and D100 of the target volume in 3D BT planning were significantly higher than in 2D BT planning (all P<0.05). For HR-CTV, the D50 in 3D BT planning was significantly higher, while D100 was lower (both P<0.05). For IR-CTV, the D95 and D100 in 3D BT planning were significantly lower while V100 was higher (all P<0.05) (Table 4). In addition, in 3D planning, the dosages to the adjacent rectum and bladder were generally significantly higher but those to sigmoid colon were significantly lower than in 2D planning (P<0.05) (Table 4).

**Table 4:** Dosimetric comparison between 2D vs 3D BT planning for tumors invading adjacent tissues (n = 7).

**Target**	**BT planning**	**D50 (%)**	**D90 (%)**	**D95 (%)**	**D100 (%)**	**V100 (cm^3^)**
**GTV**	**2D planning**	140.57±1.54	104.19±1.13	98.63±0.93	88.77±0.92	0.80±0.00
	**3D planning**	213.04±2.21*	132.62±1.44*	121.53±1.24*	103.9±1.13*	0.85±0.00
**HR-CTV**	**2D planning**	158.37±1.61	104.77±1.15	93.78±0.96	69.2±0.72	28.51±0.32
	**3D planning**	257.77±2.62*	117.32±1.21	98.13±1.02	60.56±0.69*	29.22±0.31
**IR-CTV**	**2D planning**	108.61±1.13	66.15±0.71	59.61±0.66	44.11±0.51	58.87±0.64
	**3D planning**	121.8±1.32	61.32±0.65	53.14±0.58*	36.05±0.39*	67.34±0.73*
	**Volume (cm**^**3**^**)**	**BT planning**	**D1cm**^**3**^ **(Gy)**	**D2cm**^**3**^ **(Gy)**	**D5cm**^**3**^ **(Gy)**	**Dmax(Gy)**
**Rectum**	**38.00 cm**^**3**^	2D planning	4.24±0.04	3.75±0.04	3.06±0.03	6.36±0.06
		3D planning	5.061±0.05*	4.25±0.04*	3.24±0.03	8.88±0.09*
**Bladder**	**38.62 cm**^**3**^	2D planning	4.68±0.05	4.34±0.04	3.68±0.04	6.24±0.06
		3D planning	6.64±0.06*	6.01±0.06*	4.74±0.05*	9.00±0.08*
**Sigmoid colon**	**15.94 cm**^**3**^	2D planning	4.21±0.04	3.09±0.03	2.14±0.02	7.80±0.08
		3D planning	2.24±0.02*	1.98±0.02*	1.52±0.02*	3.60±0.04*

BT: brachytherapy, 2D: 2-dimensional, 3D: 3-dimensional, GTV: gross tumor volume, HR-CTV: high-risk clinical target volume, IR-CTV: intermediate-risk clinical target volume.

*P<0.05 compared with 2D BT planning.

## Discussion

With the development of 3D imaging technique, the radiotherapy of cervical cancer is currently developing rapidly [7, 12, 18–22]. GEC-ESTRO recommended the image -guided BT with the feasibility of increasing target coverage while substantially reducing the damage to critical normal organs in patients with cervical cancer [15]. The irradiated volume was adapted to the actual shape and location of target tumor, and meanwhile the classical pear-shaped isodose was adjusted to conform to the actual shape, location and size of tumor, by which OARs were thus avoided [7]. Therefore, it has been proved that 3D BT planning better delivers dosages to the target than 2D BT planning, which ensures a local control probability of more than 90% in particular for advanced disease [17, 23]. MRI has clearly been demonstrated to have advantages on any other imaging procedures in cervical cancer, allowing an accurate definition of tumors. 3D MRI-based BT planning optimization has been proved to significantly improve the dose coverage and dosimetric parameters of tumors and have excellent pelvic control [12, 17, 23–25]. This study showed in small tumors, DVH parameters for most of the GTV, HR-CTV, IR-CTV and OARs did not exhibit significant difference between 2D and 3D BT planning. While in big tumors, almost all DVH parameters for GTV, HR-CTV, IR-CTV (except D50 for GTV and D100 for IR-CTV) were significantly higher in 3D planning than in 2D planning, and dosages to OARs were also higher at different extent in 3D BT planning. This result suggests that in contrast to those studies previously reported (12, 17, 23–25), the 3D MRI BT planning exhibits advantages over 2D BT planning in a complex way. Only in big tumors does 3D BT planning show obvious advantage in increasing the target dose coverage while at the cost of increasing the dosages to adjacent OARs. Interestingly, 3D MRI BT planning has no obvious advantages over 2D planning for small cervical tumors. Further study with larger sample size is needed to validate this result.

2D point A-based BT and 3D MRI BT planning have different effects on small and big cervical tumors. It was reported that 2D BT planning achieved lower HR-CTV and IR-CTV and dosages to OARs in patients with big cervical tumors than in those with small tumors [12]. Consistently, our study showed that in 2D BT planning, DVH parameters for HR-CTV and IR-CTV in big tumors were generally significantly lower than in small tumors. In addition, there was almost no significant difference in dosages to OARs (except Dmax to sigmoid colon) between big and small tumors in our study, which is inconsistent with Tanderup’s result [12]. In 3D MRI-based BT planning, HR-CTV and IR-CTV were lower but dosages to OARs were higher in patients with big cervical tumors than in those with small tumors [12]. The present study showed that in contrast to Tanderup’s result [12], 3D BT planning achieved significantly higher target dose coverage in big cervical tumors than in small tumors. We also found that 3D BT planning achieved higher dosages to OARs in big cervical tumors at different extent than in small tumors, which is generally consistent with Tanderup’s result [12]. This result indicates the complicated response of big versus small cervical tumors to 2D and 3D BT planning, respectively. Similarly, further study with more samples will be carried out to validate this result.

For the special anatomical structure, the eccentric tumors usually receive inadequate doses. In this study, we showed that in the eccentric tumors, almost all DVH parameters for GTV, HR-CTV and IR-CTV (except D100 and V100 for GTV and D95 for IR-CTV) were significantly higher in 3D planning than in 2D planning, and dosages to bladder and sigmoid colon were all significantly lower in 3D planning. This indicates that 3D BT planning more easily makes the eccentric tumor targets achieve the required dose but reduces dosages to the OARs compared with 2D BT planning, which has not been previously reported.

In patients with cervical cancer invading adjacent tissues, our result showed the target dose coverage in 3D BT planning was generally significantly higher than in 2D BT planning, suggesting that the cervical tumors invading adjacent tissues get more irradiation in 3D BT planning. In the meanwhile, in 3D planning the dosages to adjacent rectum and bladder were significantly higher but those to sigmoid colon were lower than in 2D planning, indicating the complexity of the prescription dosages to these tumors invading adjacent tissues. Similarly, this result has not been previously reported. Next study with larger sample size will be carried out to verify the variation of the dosages to different adjacent tissues in patients with cervical cancer invading adjacent tissues in 3D MRI vs 2D BT planning.

Although MRI-guided BT planning for cervical cancer has previously been proven to have better target coverage and less irradiation to OARs than 2D planning, the detailed analysis based on the tumor size, morphology characters, invasion into the adjacent organs, etc. still remains unclear. The present study is highlighted mainly for analyzing the dosimetric difference between 3D MRI-guided and 2D point A-based BT planning in eccentric cervical tumors and tumors invading adjacent tissues. In addition, we showed 3D MRI BT planning exhibited advantages over 2D planning only for big tumors instead of small tumors. We also revealed the complex response of OARs to 2D and 3D BT planning, respectively in cases with small vs big cervical tumors. All these aspects above have been rarely previously reported.

## Conclusion

This study indicates that 3D MRI image-guided BT planning generally increases the tumor dose coverage and dosages to OARs in cases with big tumors in comparison with 2D point A-based BT planning. The response of small versus big cervical tumors to 2D and 3D BT planning is diverse. In the eccentric cervical tumors, 3D BT planning makes the eccentric tumors achieve more required dosage and reduces dosages to the OARs than 2D BT planning. In cervical tumors invading adjacent tissues, 3D planning generally increases the tumor dose coverage but diversely affects the dosages to OARs compared with 2D planning. This study comprehensively indicates that 3D MRI image-guided BT planning exhibits advantages over 2D point A-based BT planning in a complex way, generally showing advantages for the treatment of cervical cancer except small tumors. This study will be beneficial to the decision-making of the BT planning for the treatment of cervical cancer.

## Supporting Information

S1 Table Dosimetric comparison between small (n = 59) and big tumors (n = 20) in 2D and 3D planning, respectively. (DOC)
