# Supplementary material for: Dosimetric Comparison between Three-Dimensional Magnetic Resonance Imaging-Guided and Conventional Two-Dimensional Point A-Based Intracavitary Brachytherapy Planning for Cervical Cancer
Source: PLoS One. 2016 Sep 9;11(9):e0161932. doi: 10.1371/journal.pone.0161932 (PMC5017728; doi:10.1371/journal.pone.0161932)
Supplement: S1 Table — (DOC) [file pone.0161932.s001.doc]

**S1 Table. Dosimetric comparison between small (n=59) and big tumors (n=20) in 2D and 3D planning, respectively**

|  | **D50 (%)** | **D90 (%)** | **D95 (%)** | **D100 (%)** | |
| --- | --- | --- | --- | --- | --- |
| **3D planning-GTV** | | | | | |
| **Small** | 242.85±2.31 | 190.20±1.82 | 176.25±1.63 | 156.34±1.49 | |
| **Big** | 263.31±2.51* | 218.49±2.07* | 209.19±2.01* | 185.21±1.76* | |
| **3D planning-HR-CTV** | | | | | |
| **Small** | 213.54±2.02 | 112.93±1.02 | 98.87±0.94 | 66.34±0.48 | |
| **Big** | 227.03±2.15* | 121.27±1.02* | 104.61±1.01* | 72.76±0.50* | |
| **3D planning-IR-CTV** | | | | | |
| **Small** | 119.29±1.01 | 60.66±0.53 | 54.14±0.41 | 38.34±0.27 | |
| **Big** | 129.08±1.15* | 69.16±0.51* | 61.44±0.41* | 44.15±0.31* | |
| **3D planning** | **D0.1cm^3^ (Gy)** | **D1cm^3^ (Gy)** | **D2cm^3^ (Gy)** | **D5cm^3^ (Gy)** | **Dmax(Gy)** |
| **3D planning-sigmoid colon** | |  |  |  |  |
| **Small** | 4.46±0.00 | 3.91±0.00 | 3.65±0.00 | 3.24±0.00 | 5.02±0.00 |
| **Big** | 6.42±0.00* | 5.40±0.00 | 5.05±0.00* | 4.42±0.00 | 7.31±0.01* |
| **3D planning--rectum** | | | | | |
| **Small** | 4.29±0.00 | 3.71±0.00 | 3.47±0.00 | 3.10±0.00 | 4.88±0.01 |
| **Big** | 5.52±0.01* | 4.71±0.01 | 4.37±0.00 | 3.85±0.00 | 6.33±0.01* |
| **3D planning--bladder** | | | | | |
| **Small** | 5.52±0.00 | 4.78±0.01 | 4.43±0.00 | 3.84±0.00 | 6.13±0.01 |
| **Big** | 6.08±0.01* | 5.17±0.00 | 4.79±0.00 | 4.19±0.00 | 6.81±0.01* |
|  | **D50 (%)** | **D90 (%)** | **D95 (%)** | **D100 (%)** | |
| **2D planning-GTV** | | | | | |
| **Small** | 262.51 ±2.31 | 182.59 ±1.78 | 170.31 ±1.37 | 134.38 ±1.21 | |
| **Big** | 262.96 ±2.45 | 183.80±1.70 | 170.11±1.61 | 139.70±1.20 | |
| **2D planning-HR-CTV** | | | | | |
| **Small** | 183.39 ±1.78 | 111.04 ±1.02 | 98.58±0.89 | 68.96 ±0.49 | |
| **Big** | 165.83±1.52* | 91.47±0.89* | 80.49±0.71* | 56.68±0.41* | |
| **2D planning-IR-CTV** | | | | | |
| **Small** | 118.78 ±1.01 | 64.31 ±0.43 | 56.63 ±0.46 | 39.43±0.28 | |
| **Big** | 99.92 ± 0.91* | 56.14±0.49* | 49.71±0.36* | 35.51±0.32 | |
| **2D planning** | **D 0.1cm^3^ (Gy)** | **D1cm^3^ (Gy)** | **D2cm^3^ (Gy)** | **D5cm^3^ (Gy)** | **Dmax(Gy)** |
| **2D planning-sigmoid colon** | | | | | |
| **Small** | 4.56 ± 0.00 | 4.23 ± 0.00 | 3.99 ± 0.00 | 3.59 ± 0.00 | 5.09 ± 0.00 |
| **Big** | 4.95 ± 0.00 | 4.51 ± 0.00 | 4.21 ± 0.00 | 3.83 ± 0.00 | 5.55 ± 0.01 * |
| **2D planning-rectum** | | | | | |
| **Small** | 4.12 ± 0.00 | 3.65 ± 0.00 | 3.45 ± 0.00 | 3.12 ± 0.00 | 4.71 ± 0.01 |
| **Big** | 4.12 ± 0.01 | 3.62 ± 0.01 | 3.48 ± 0.00 | 3.13 ± 0.00 | 4.69 ± 0.01 |
| **2D planning-bladder** | | | | | |
| **Small** | 5.34 ± 0.00 | 4.78 ± 0.01 | 4.35 ± 0.00 | 4.03 ± 0.00 | 5.90 ± 0.01 |
| **Big** | 5.39 ± 0.01 | 4.75 ± 0.00 | 4.32 ± 0.00 | 4.02 ± 0.00 | 5.87 ± 0.01 |

2D: 2-dimensional, 3D: 3-dimensional, BT: brachytherapy, GTV: gross tumor volume, HR-CTV: high-risk clinical target volume, IR-CTV: intermediate-risk clinical target volume. *P<0.05 compared with small tumors.
